# Pressure pain sensitivity map of multifocal nummular headache: a case report

**DOI:** 10.1186/s10194-015-0523-7

**Published:** 2015-04-30

**Authors:** Cristina Rodríguez, Sonia Herrero-Velázquez, Marina Ruiz, Johanna Barón, Alicia Carreres, Edert Rodríguez-Valencia, Angel Luis Guerrero, Pascal Madeleine, María Luz Cuadrado, César Fernández-de-las-Peñas

**Affiliations:** Neurology Department, Hospital Clínico Universitario de Valladolid, Avda. Ramón y Cajal 3, 47005 Valladolid, Spain; Neurology Department, Hospital Universitario Rio Hortega de Valladolid, Valladolid, Spain; Department of Health Science and Technology, Physical Activity and Human Performance Group, SMI, Aalborg University, Aalborg, Denmark; Neurology Department, Hospital Clínico San Carlos, Universidad Complutense de Madrid, Madrid, Spain; Departamento de Fisioterapia, Terapia Ocupacional, Rehabilitación y Medicina Física, Universidad Rey Juan Carlos, Alcorcón, Madrid

**Keywords:** Algometry, Gabapentin, Nummular headache, Multifocal, Pressure pain thresholds

## Abstract

**Background:**

Nummular headache (NH) is most commonly a localized unifocal headache; however, some patients infrequently exhibit multifocal symptomatic painful head areas retaining all features of NH. We present the pressure pain sensitivity map of an adolescent with multifocal NH.

**Case presentation:**

We describe a case of a 14 year-old-girl with a 3-year history of continuous pain in four rounded areas, all of them with the same size and shape. Pressure pain thresholds (PPT) were assessed on 21 points over the scalp and over the symptomatic areas. A pressure pain sensitivity map of the head was constructed. The neurological exam was unremarkable, with neither sensory symptoms nor trophic changes within the painful areas. As previously shown, symptomatic points exhibited lower PPTs compared to the surrounding areas. The map reflected 4 restricted areas of mechanical hyperalgesia confined just to the painful areas. Treatment with gabapentin achieved complete remission.

**Conclusion:**

This is the first pain sensitivity map of a patient with multifocal NH. Our results support peripheral mechanisms are maintained in multifocal NH.

## Background

Nummular headache (NH) was described in 2002 by Pareja et al. [1]. It has been included in the main body of the International Classification of Headache Disorders, 3^rd^ edition, beta version (ICHD-3 beta) under the category of other primary headaches [2]. NH has been characterized as a focal pain, either continuous or intermittent, which remains confined to a small round or elliptical area of the scalp. Pain intensity is generally mild or moderate, but superimposed *in situ* exacerbations are frequently described [3,4]. The painful area is usually located in the parietal scalp and commonly presents with variable combinations of hyperesthesia, dysesthesia, paresthesia or allodynia [3].

Though, NH is typically unifocal, a small number of patients have been reported with focal head pain in at least 2 separate areas [5-9]. The shape and size of the painful areas were quite similar in each particular patient, but, in contrast, spatial and temporal relations, pain intensity, exacerbations or response to therapy could differ between different areas in the same patient. For the first time, we have obtained a pressure pain sensitivity map of the scalp in a patient presenting with multifocal NH.

## Case presentation

A 14 year-old-girl presented with a 3-year history of continuous pain in four rounded areas of 4 cm in diameter, symmetrically located at the parietal and occipital regions of the scalp, all of them equal in size and shape. The pain was described as oppressive, and graded 5 out of 10 on a numerical pain rate scale (NPRS; 0: no pain, 10: the worst imaginable pain). The patient did not identify any trigger. Neurological and general examinations were unremarkable with neither sensory symptoms nor local trophic changes present within the affected regions of the head. The occipital and auriculotemporal nerves were not tender to palpation. Brain magnetic resonance imaging and routine blood work-up with erythrocyte sedimentation rate and immunological screening were obtained with no abnormalities. The patient had used acetaminophen with no substantial relief of her symptoms.

We decided to conduct a cartographic study of pressure pain sensitivity on the patient’s scalp to evaluate the presence of localized or generalized hyperalgesia. The procedure was conducted following previously published guidelines [10]. Pressure pain thresholds (PPT) were assessed on 21 points distributed over the scalp. The locations and nomenclature of these points were based on standard position of international 10/20 and 10/10 systems for electroencephalogram (EEG) recordings: eight points on the right (Fp2, F4, F8, C4, T4, P4, T6 and O2), eight points on the left (Fp1, F3, F7, C3, T3, P3, T5 and O1) and five points along the mid-sagittal curve (Fpz, Fz, Cz, Pz, and Oz). In addition, PPTs were evaluated on the 4 symptomatic areas. Thereby, the patient had 25 matching points for PPT assessments (the 21 standardized points plus the four symptomatic points). The parietal symptomatic areas were located behind C3 and C4 points, and the occipital areas were respectively located between O2-T4 and O1-T5. All these points were marked with a pen over the scalp.

PPTs were measured using a pressure algometer. This device is a 1 cm^2^ rubber disk attached to the pole of a pressure gauge, which displays pressure values in kg/cm^2^. The patient indicated verbally to stop the pressure stimulation when the threshold was reached. PPT was defined as the minimal amount of pressure where a sense of pressure first changed to pain. Three consecutive measurements at intervals of 30 seconds were obtained on each point, and then converted to kPa (SI unit) for the analysis. The order of point assessment was randomized [10].

**Figure 1:**
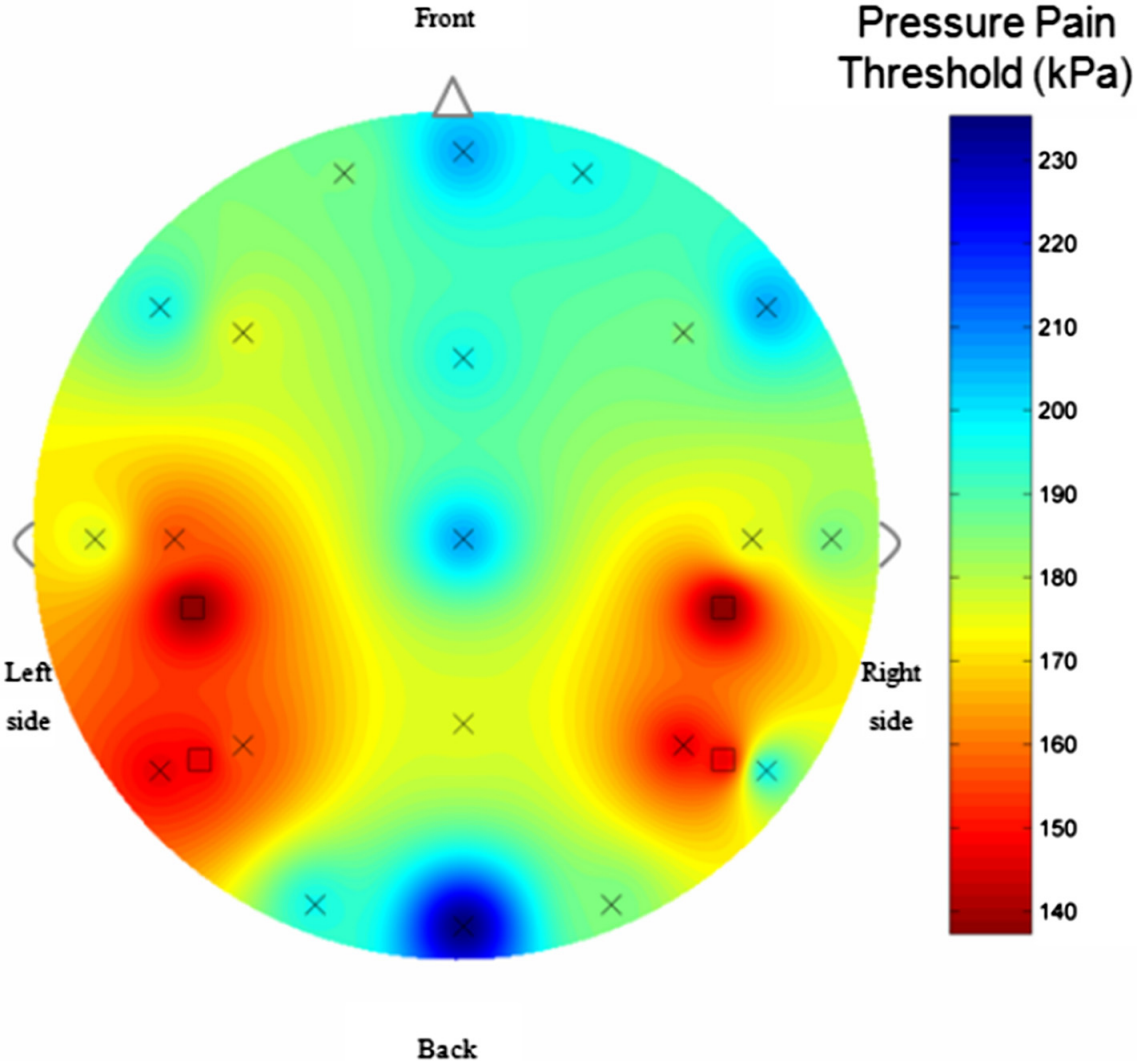
Topographical pressure pain sensitivity map of the case report with multifocal nummular headache.

The results of PPT measurements can be observed on Table 1. PPT data were used to construct a map of the spatial distribution of pressure pain sensitivity over the scalp with appropriate software applications [10]. Figure 1 graphs the pressure pain sensitivity map of the scalp. As previously shown in NH [11], symptomatic points had lower PPT values than the surrounding scalp areas. Therefore, the map clearly showed four patches of hyperalgesia at the painful zones (Figure 1). Preventive treatment with 900 mg/day of gabapentin achieved complete pain remission at short and long-term follow-ups (20 months).

**Table 1:** **Mean pressure pain thresholds (PPT, kPa) over the scalp**

**Localization**	**Mean PPT**
Fpz	205.9
Fp1	186.3
Fp2	192.2
Fz	106.1
F3	176.5
F4	186.3
F7	166.7
F8	205.9
CZ	205.9
C3	156.9
C4	176.5
T3	166.7
T4	186.3
PZ	176.5
P3	156.9
P4	147.1
T5	147.1
T6	186.3
OZ	225.6
O1	106.1
O2	186.3
Painful point behind C4	147.1
Painful point behind C3	147.1
Painful point O2-T4	147.1
Painful point O1-T5	147.1

### Discussion

This case report fits diagnostic criteria for NH according to ICHD-III. Multifocal pain, though rarely described in the literature, is in fact mentioned in the classification system [2]. In multifocal NH, shape and size of symptomatic areas are identical, as in our patient, or quite similar. There are more differences when considering spatial relationship between the painful areas since the most frequently observed pattern is pain over symmetrical points on both sides of the scalp [6]. Yet, there are also reports of pain in asymmetrical points on each side [7], and location of painful areas on the same side of the head [6,8,12]. Regarding the temporal sequence of pain felt in different areas, it may be synchronous, additive, or even migratory [6-8].

Confinement of symptoms observed in NH suggests that pain stems from terminal branches of sensory nerves located in epicranial tissues [1]. Nevertheless, appearance of multifocal NH reports could encourage a discussion regarding its pathogenesis, as they may correspond to either a generalized disorder or a peripheral dysfunction occurring in multiple areas. Both the absence of trophic changes and the response to low doses of gabapentin make it unlikely to be the presence of a generalized skin disorder in our patient.

Pressure algometry has good reliability and is considered a useful tool for research on headache pathophysiology [11,13]. In previous studies in NH, only the symptomatic zone had shown local decreases of the pain thresholds to pressure, with a characteristic appearance in the map of sensitivity [10], in our study the map showed four patches of hyperalgesia at the painful zones, and the symptomatic points had lower PPT values than the surrounding scalp areas, as previously shown in NH [11].

## Conclusion

To the best of the author’s knowledge, this is the first pressure pain sensitivity map of multifocal NH in the scientific literature. According to our results, peripheral mechanisms seem to be maintained within each painful area as our patient did not show widespread hyperalgesia. Instead, she had separate patches of hyperalgesia corresponding to local decreases of PPT in each of the painful areas.

## Consent

Written informed consent was obtained from the patient for publication of this Case report and any accompanying images. A copy of the written consent is available for review by the Editor-in-Chief of this journal.
